# Magnetic compression anastomosis for the complete dehiscence of hepaticojejunostomy in a patient after living-donor liver transplantation

**DOI:** 10.1186/s40792-018-0504-6

**Published:** 2018-08-15

**Authors:** Masahiko Kubo, Hiroshi Wada, Hidetoshi Eguchi, Kunihito Gotoh, Yoshifumi Iwagami, Daisaku Yamada, Hirofumi Akita, Tadafumi Asaoka, Takehiro Noda, Shogo Kobayashi, Masahisa Nakamura, Yusuke Ono, Keigo Osuga, Eigoro Yamanouchi, Yuichiro Doki, Masaki Mori

**Affiliations:** 1 Department of Gastroenterological Surgery, Graduated School of Medicine, Osaka University Hospital, 2-2-E2, Yamadaoka, Suita city, Osaka prefecture 565-0871 Japan; 2 Department of Diagnostic and Interventional Radiology, Graduated School of Medicine, Osaka University, Suita city, Osaka prefecture Japan; 3 Department of Radiology, International University of Health and Welfare Hospital, Nasushiobara city, Tochigi prefecture Japan

**Keywords:** Magnetic compression anastomosis, Living-donor liver transplantation, Complete dehiscence of bilioenteric anastomosis

## Abstract

**Background:**

Magnetic compression anastomosis (MCA) is a minimally invasive method of anastomosis that does not involve a surgical procedure in patients with stricture, obstruction, or dehiscence of anastomosis after surgery. We experienced a case of complete dehiscence of bilioenteric anastomosis that was successfully treated by MCA.

**Case presentation:**

A 55-year-old woman received ABO-incompatible right-lobe living-donor liver transplantation with hepaticojejunostomy for the right anterior duct (RAD) and right posterior duct (RPD). Nineteen days after the operation, bilious and bloody discharge was detected from the abdominal drain. We performed an emergency operation and found that the anastomosis was completely dehiscent. We placed bile drainage catheters into the stumps of the RAD and RPD. She repeatedly experienced cholangitis after the surgery, so we added percutaneous transhepatic cholangial drainage (PTCD) tubes. We decided to treat the complete dehiscence of anastomosis by MCA. One year after the liver transplantation, we performed MCA for the RAD. The bilioenteric fistula was completed 21 days after MCA, and the magnets were retrieved by double-balloon endoscopy. Two months later, MCA for the RPD was also performed by the same procedure. The bilioenteric fistula was not completely established, so we performed double-balloon endoscopy and pulled the magnets down 47 days after MCA for the RAD. The internal/external bile drainage tubes were then left in place to maintain the bilioenteric fistula. Twelve months after MCA for the RAD and 19 months after MCA for the RPD, we removed the tubes without any complications.

**Conclusion:**

Magnetic compression anastomosis for stricture, obstruction, or dehiscence of the anastomosis after living-donor liver transplantation was an effective and safe procedure.

## Background

Despite improvements in surgical techniques and the postoperative management of liver transplantation, complications of biliary reconstruction are still common and can influence the postoperative graft survival after transplantation. For biliary stricture, balloon dilation or the placement of a plastic stent under percutaneous transhepatic cholangiography (PTC) or endoscopic retrograde cholangiography (ERC) is generally performed. However, it is difficult to resolve complete stenosis or dehiscence of biliary anastomosis by PTC or ERC. In such cases, re-anastomosis is a treatment option, but the surgical procedures for re-anastomosis are invasive and difficult to perform because of postoperative adhesion and patients’ general condition.

Magnetic compression anastomosis (MCA) is a relatively safe method of anastomosis that does not require surgery in patients with stricture, obstruction, or dehiscence of anastomosis after surgery.

We herein report a patient with complete dehiscence of bilioenteric anastomosis after living-donor liver transplantation (LDLT) that was successfully treated by MCA.

## Case presentation

The patient was a 55-year-old Japanese woman with decompensated cirrhosis by primary sclerosing cholangitis. Her Child-Pugh score was 13 points, which was categorized as class C, and the model for end-stage liver disease (MELD) score was 23 points. She underwent ABO-incompatible LDLT by right-lobe graft with hepaticojejunostomy for the right anterior duct (RAD) and right posterior duct (RPD). We performed hepaticojejunostomy using the “open-up” anastomotic technique, as described previously [1]. In brief, both the anterior and posterior walls of the graft bile duct were opened using 6-0 absorbable monofilament sutures before anastomosis. The graft bile ducts were anastomosed to the recipient jejunum by interrupted sutures, and a biliary drainage tube was placed for each bile duct across the site of anastomosis and exteriorized by the Witzel procedure.

**Fig. 1:**
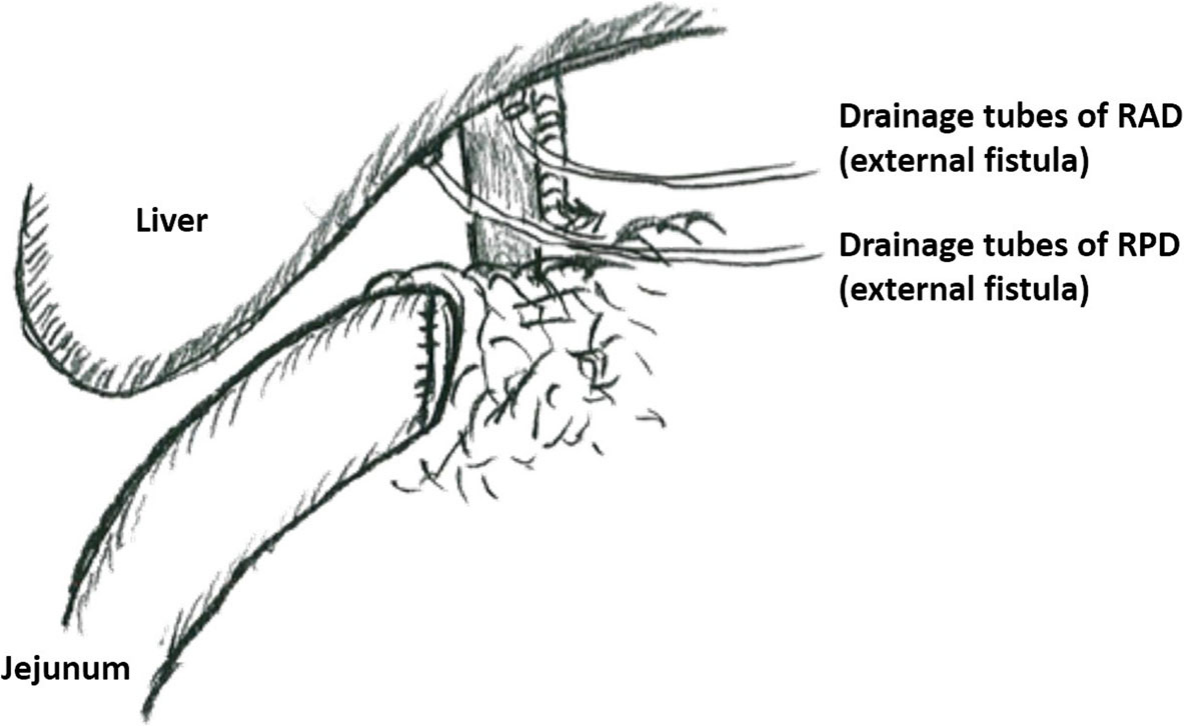
In redo surgery, biliary drainage tubes that were inserted from the stumps of the RAD and PRD were exteriorized through the abdominal free space and abdominal wall. The jejunum was simply placed and fixed near the RAD and RPD for future anastomosis by MCA. *RAD* right anterior duct, *RPD* right posterior duct

Eighteen days after LDLT, double-balloon endoscopy was performed for bleeding in the digestive tract. Nineteen days after LDLT, bilious and bloody discharge was detected from the abdominal drain, and we performed emergency surgery. In the operation, we found that the hepaticojejunostomy of both the RAD and RPD suffered complete dehiscence, and re-anastomosis was impossible. Thus, we decided to avoid re-anastomosis of the bile ducts in the operation. Biliary drainage tubes were inserted from the stumps of the RAD and PRD and were exteriorized through the abdominal free space and abdominal wall. We avoided inserting biliary drainage tubes transhepatically due to the possibility of injuring the graft liver. The jejunum was simply placed and fixed near the RAD and RPD for future anastomosis by MCA (Fig. 1). After surgery, we added percutaneous transhepatic cholangiography drainage (PTCD) tubes for the RAD and RPD to reduce the frequency of cholangitis. Her general condition gradually improved, and she was discharged 6 months after LDLT.

**Fig. 2:**
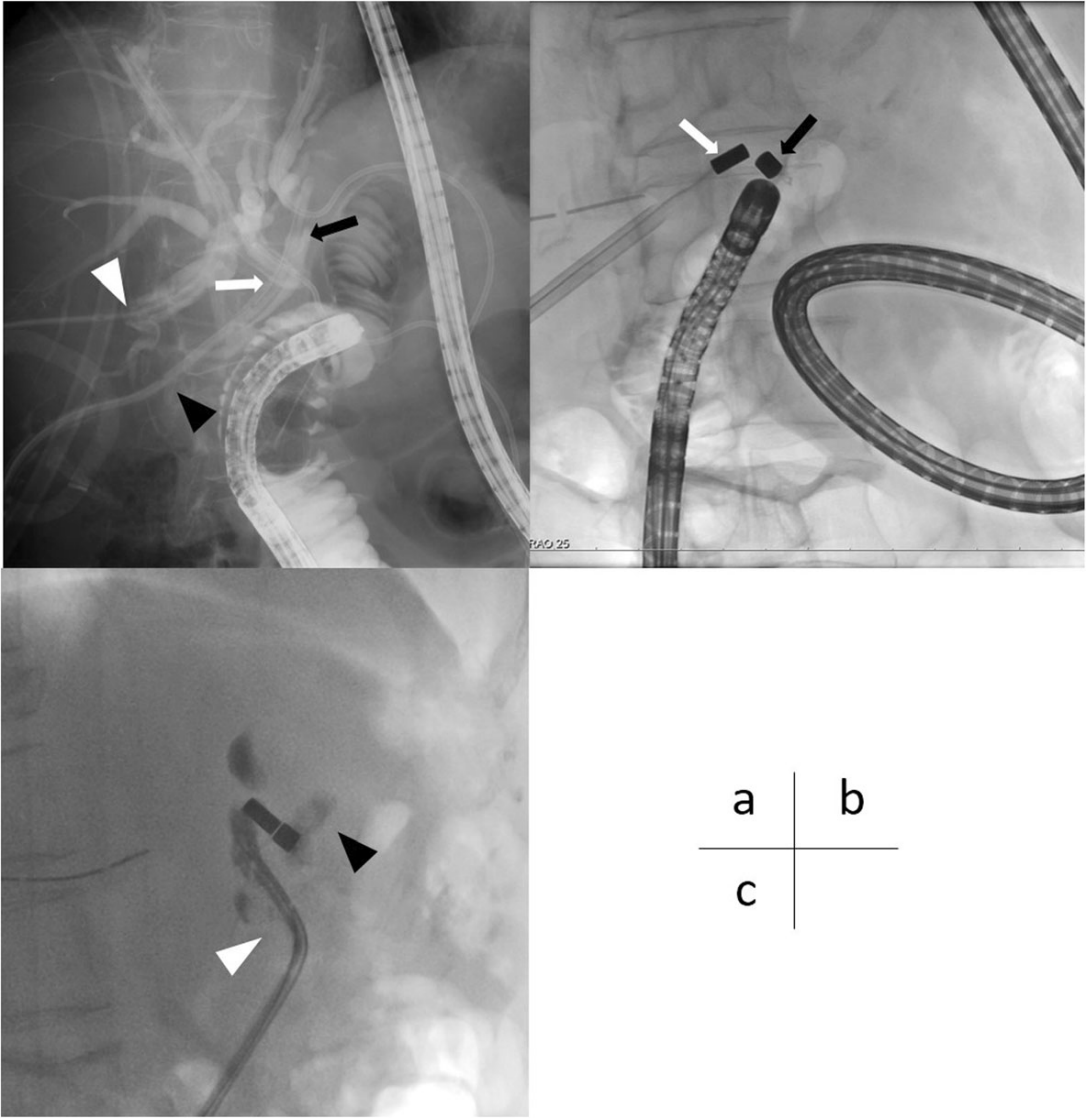
**a** We checked that the stumps of the RAD (black arrow) and RPD (white arrow) were near the bowel for MCA by contrast radiography. We placed PTCD tubes in the RAD (black arrowhead) and RPD (white arrowhead). **b** MCA for the RAD was performed. The magnet (black arrow) was introduced into the bowel near the RAD by double-balloon endoscopy, and another magnet (white arrow) was inserted through PTCD of the RAD. **c** We confirmed the outflow of the contrast agent into the bowel (black arrowhead) via the PTCD tube placed in the RAD (white arrowhead). The bilioenteric fistula of the RAD was completed 21 days after MCA. *MCA* magnetic compression anastomosis, *RAD* right anterior duct, *RPD* right posterior duct

**Fig. 3:**
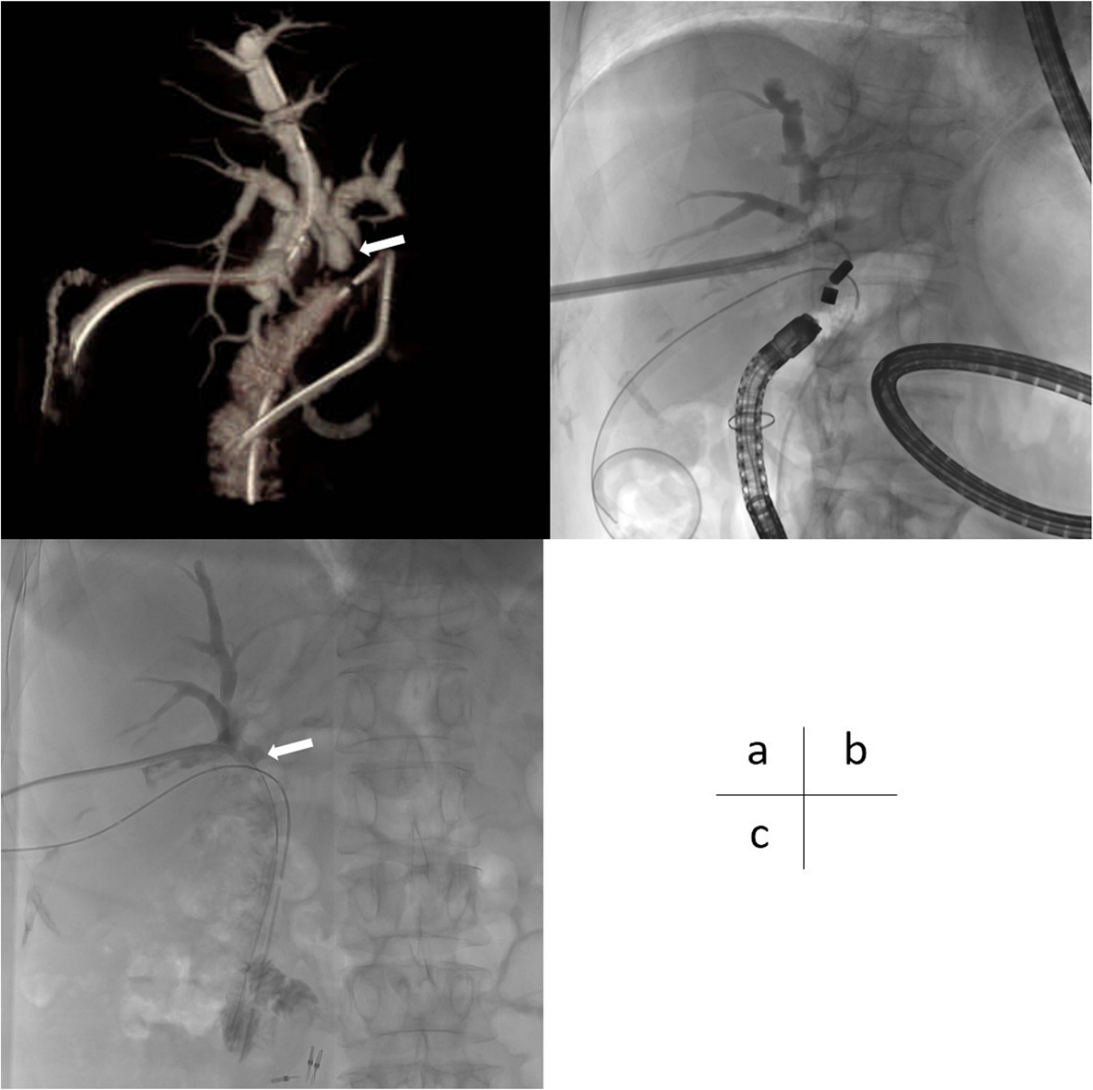
**a** We checked the stumps of the RPD (white arrow) by 3D reconstruction of CT image. **b** MCA for the RPD was performed by the same procedure as that for the RAD. **c** The bilioenteric fistula of the RPD was completed 47 days after MCA. The PTCD tube was introduced into the bowel to maintain the fistula through re-anastomosis of RPD (white arrow). *MCA* magnetic compression anastomosis, *RAD* right anterior duct, *RPD* right posterior duct

We next decided to treat the complete dehiscence of bilioenteric anastomosis by MCA and consulted with Professor Yamanouchi about MCA in order to improve the quality of life (QOL) and control her cholangitis. One year after LDLT, we performed contrast radiography using double-balloon endoscopy and the PTCD tubes to measure the distance between the stumps of the RAD and RPD and bowel (Fig. 2a). The magnet was introduced into the bowel and placed near the RAD by double-balloon endoscopy. We replaced the 16 Fr silicon PTCD tube with a 14-Fr introducer sheath. The other magnet was inserted through the introducer sheath (Fig. 2b). At the end of this procedure, we replaced the introducer sheath with a 14-Fr silicon tube. The magnets that were used for MCA are not yet commercially available in Japan. The bilioenteric fistula was completed 21 days after MCA (Fig. 2c), and we removed the magnets by double-balloon endoscopy. The PTCD tube of the RAD was inserted into the bowel and left to maintain the fistula. Two months later, MCA for the RPD was also performed by the same procedure. Before the procedure, we confirmed the RPD’s structure by 3D reconstruction of CT image (Fig. 3a) to check that there were no obstacles, such as bilioenteric anastomosis of the RAD between the stump of the RPD and bowel. It took longer to establish the RPD’s bilioenteric fistula than that of the RAD because the angle of the RPD’s structure was sharp and the force between the magnets was weak (Fig. 3b). Therefore, we adjusted the position of magnet in the bile duct and its wire several times.

Forty-seven days after performing MCA of the RPD, we performed double-balloon endoscopy and pulled down the magnets. We confirmed that the fistula of the RPD had been established and retrieved the magnets. The PTCD tube of the RPD was also introduced into the bowel to maintain the fistula (Fig. 3c). One year after MCA of the RAD, we confirmed the complete establishment of bilioenteric anastomosis for the RAD and removed the PTCD tube in the RAD.

With the confirmation of the complete establishment of anastomosis without stricture, we removed the PTCD tube in the RPD 19 months after MCA of the RPD. The patient is still alive without any tubes, and no complications, such as stricture, have occurred.

## Discussion

LDLT has been performed in Japan since 1993 [2] and is the most effective procedure in patients with end-stage liver disease and acute liver failure. Recent advancements in surgical techniques and postoperative management have resulted in an excellent graft survival rate of over 90% [3]. However, post-LDLT biliary complications are still a major problem.

The biliary complications are mainly biliary anastomotic stricture (BAS) or biliary leakage (BL), with incidences of 20.2% and 9.2%, respectively [4]. Several approaches to resolving these complications have been reported, such as endoscopic techniques, percutaneous transhepatic intervention, and surgical procedures [5]. The conventional approach to addressing biliary complications is a surgical procedure. However, surgical re-anastomosis for BAS is difficult and has a high morbidity rate [6]. Nonsurgical techniques are now the first choice for managing biliary complications. Ballooning or inserting plastic stents into biliary anastomosis via ERC or PTC are the main nonsurgical techniques. These treatments’ success rates are reported to range from 60 to 84% for ERC and 40 to 85% for PTC, making them the gold-standard methods for managing biliary complications after LDLT [7]. However, neither ERC nor PTC can treat complete dehiscence of biliary anastomosis, because the guidewire cannot pass through the site of anastomosis in many cases. A new non-surgical approach for dehiscence or complete stenosis instead of surgical re-anastomosis is therefore needed. Recently, the endoscopic ultrasonography (EUS)-guided biliary drainage has emerged as a promising modality in patients in whom ERC fails [8]. EUS-guided hepaticoenterostomy is mainly performed with the puncture of a branch of the intrahepatic duct in the lateral segment of the liver. A few reports have shown successful biliary drainage of the right hepatic lobe by EUS-guided hepaticoenterostomy. Although these EUS-guided procedures are mainly performed in cases involving malignant disease, EUS-guided hepaticoenterostomy would be a treatment option for complete obstruction or dehiscence of biliary anastomosis in the field of liver transplantation.

MCA is a relatively safe procedure for reconstruction of entericoenteric, biliobiliary, or bilioenteric anastomosis complications. MCA involves placing two magnets into the gastrointestinal tract or bile duct and making anastomosis by magnetic pressure without surgery. This innovative method was invented by Dr. Yamanouchi in the 1990s [9] and has been performed to resolve several complications of anastomosis, such as hepaticojejunostomy leakage after surgery for cholangiocarcinoma, esophagojejunostomy obstruction after total gastrectomy, or ileus by cancerous peritonitis [10–12]. The magnets are not yet commercially available in Japan. MCA is also performed after LDLT, and 40 cases of MCA for biliary complications after LDLT have been reported so far (Table 1).

**Table 1:** Characteristics and outcomes of patients who underwent magnetic compression anastomosis for biliary complications after living-donor liver transplantation

Years	Author	No.	Age (years)	Sex	Disease	Reconstruction	Biliary complication	Intervention	Distance (mm)	Duration from LDLT (months)	Period until removal of magnet (days)	Duration of internal catheter (weeks)	Outcome
2005	Muraoka et al. [15]	1	1	F	FH	R-Y	Stricture	CJS	N/A	6	12	8	Success
		2	57	M	LC	R-Y	Stricture	CJS	N/A	7	12	8	Success
2005	Okajima et al. [16]	3	44	F	FH	D-D	Obstruction	CCS	N/A	12	42	12	Success
2008	Akita et al. [17]	4	34	F	N/A	D-D	Obstruction	CCS	5	0	21	0	Success
2008	Mita et al. [18]	5	0.5	F	FH	R-Y	Obstruction	CJS	N/A	1.5	12	N/A	Success
		6	59	F	LC	R-Y	Obstruction	CJS	N/A	5	25	N/A	Success
2009	Matsuno et al. [19]	7	53	M	N/A	D-D	Stricture	CCS	3	N/A	10	4.3	Success
2010	Marubashi et al. [13]	8	53	M	LC (HBV)	D-D	Dehiscence	CCS	N/A	6	63	72	Success
2010	Itoi et al. [20]	9	60	M	N/A	D-D	Obstruction	CCS	N/A	18	9	24	Success
2011	Kawakubo et al. [6]	10	56	M	LC (HCV)	R-Y	Obstruction	CJS	N/A	9	23	56	Success
2011	Jang et al. [14]	11	63	F	LC (HBV)	D-D	Stricture	CCS	N/A	71	42	83	Success
		12	49	M	LC (HBV)	D-D	Stricture	CCS	N/A	6	26	70	Success
		13	54	M	LC (HBV)	D-D	Stricture	CCS	N/A	55	18	49	Success
		14	64	F	LC (HBV)	D-D	Stricture	CCS	N/A	71	71	14	Success
		15	54	M	LC (HBV)	D-D	Stricture	CCS	N/A	15	102	26.7	Success
		16	48	M	LC (HBV)	D-D	Stricture	CCS	N/A	13	102	25.7	Success
		17	63	F	Liver failure (drug)	D-D	Stricture	CCS	30	107	–	–	Failure
		18	33	M	Liver failure (HAV)	D-D	Stricture	CCS	N/A	5	33	26.7	Success
		19	61	M	LC (HBV)	D-D	Stricture	CCS	N/A	4	14	25.4	Success
		20	54	M	LC (HBV)	D-D	Stricture	CCS	N/A	9	181	7.3	Success
		21	52	M	LC (HBV)	D-D	Stricture	CCS	N/A	1	153	N/A	Success
		22	51	M	LC (HBV)	D-D	Stricture	CCS	N/A	5	–	–	Failure
2014	Uemura et al. [21]	23	49	F	LC (HCV)	D-D	Obstruction	CCS	11	11	24	13	Success
2016	Ersoz et al. [22]	24	37	M	LC (HBV)	D-D	Dehiscence	CCS	15	12	13	N/A	Success
		25	54	F	LC (HBV, HDV)	D-D	Dehiscence	CCS	10	84	14	N/A	Success
		26	53	F	LC (HCV)	D-D	Dehiscence	CCS	10	14	42	N/A	Success
		27	63	M	LC (HBV)	D-D	Dehiscence	CCS	5	7	18	N/A	Success
		28	68	F	LC (HBV)	D-D	Dehiscence	CCS	10	6	22	N/A	Success
		29	54	M	LC (HBV)	D-D	Dehiscence	CCS	15	7	13	N/A	Success
2017	Saito et al. [23]	30	54	F	LC (PSC)	R-Y	Strangulated ileus	CDS	27	60	25	44	Success
2017	Parlak et al. [24]	31	68	F	LC (HCV)	D-D	Obstruction	CCS	2.5	14	7	36	Success
		32	52	M	LC (HBV)	D-D	Obstruction	CCS	3	22	10	N/A	Success
		33	55	M	LC (HBV)	D-D	Obstruction	CCS	4	16	2	36	Success
		34	58	M	LC (Cryptogenic)	D-D	Obstruction	CCS	6	34	14	48	Success
		35	38	F	LC (HBV)	D-D	Obstruction	CCS	3	38	12	48	Success
		36	63	F	LC (HCV)	D-D	Obstruction	CCS	3	13	2	44	Success
		37	52	M	LC (HBV)	D-D	Obstruction	CCS	4	60	10	N/A	Success
		38	54	M	LC (HBV)	D-D	Obstruction	CCS	5	15	–	–	Failure
		39	62	M	LC (Cryptogenic)	D-D	Obstruction	CCS	5	5	–	–	Failure
2018	Kubo	40	55	F	LC (PSC)	R-Y	Dehiscence	CJS	10	12	21	48	Success
							Dehiscence	CJS	10	14	47	76	Success
	Mean		51.6		s				8.9	22.6	35.5	37.6	36/40(90.0%)

*FH* fulminant hepatitis, *LC* liver cirrhosis, *HAV* hepatitis type A virus, *HBV* hepatitis type B virus, *HCV* hepatitis type C virus, *PSC* primary sclerosing cholangitis, *D-D* duct-to-duct anastomosis, *R-Y* Roux en Y anastomosis, *CCS* choledochocholedochostomy, *CDS* choledochoduodenostomy, *CJS* choledochojejunostomy, *N/A* not addressed

The success rate of MCA for biliary complication after LDLT was 36/40 (90.0%). Only one case of 40 (2.5%) experienced restenosis. The reasons for failure included the common bile duct being too narrow and tortuous to insert the magnet in one case, the stricture distance being too long in one case, and the angle between the magnets being too wide in two cases. To our knowledge, this is the first report of MCA for two bilioenteric anastomoses after LDLT. In our case, the period from inserting magnets to their removal in MCA for the RAD was shorted than the mean (35 days). However, the period with MCA for the RPD was longer than that with MCA for the RAD. We believe that the angle of the RPD is one of the main reasons for the delay of anastomosis by MCA. In our case, the angle was too sharp to achieve the appropriate pressure with the magnet in bile duct.

The mean distance between the two magnets in the 40 cases was 8.9 mm. The distance between the magnets should be kept below 20 mm, because of the magnets’ power [13, 14]. The distance in almost all cases (except for 2) was under 20 mm. One case of MCA with a distance of 27 mm succeeded, while the other with a distance of 30 mm failed. While the magnets’ distance is recommended to be kept under 20 mm, further discussion with the accumulation of more cases is needed. In our case, the magnets’ distances in the RAD and RPD were 10 mm, which should be appropriate for MCA.

According to our search of PubMed, complications associated with MCA performed to resolve post-LDLT biliary complications, such as bleeding or perforation, have not been reported. One case of restenosis of the common bile duct after MCA was reported [14]. In this case, a guide wire was passed through the stricture easily and an internal drainage catheter was inserted. The catheter was removed 4 months after the treatment, and no further restenosis occurred. The complication rate of MCA was lower than that of surgical re-anastomosis.

One issue associated with MCA is the long time required to complete the treatment. To maintain the anastomotic fistulas, a catheter or stent is inserted for several months after MCA. In the 40 cases of MCA for biliary complications after LDLT, the mean treatment duration was 9.4 months. The duration of dehiscence (16.3 months) tended to be longer than the duration of stricture (7.3 months) or obstruction (9.8 months). In our case, the treatment durations were 12 months in the RAD and 19 months in the RPD. We performed contrast-enhanced radiography via the catheters and confirmed the fistulas every 3 months. It takes longer to remove the tube in the RPD than that in the RAD because the angle of anastomosis was sharp and resembled stricture. Fortunately, no complications occurred after removing the catheters. Our experience suggests that careful examinations are necessary when removing catheters after MCA.

Despite these issues, MCA is a less-invasive, safe, effective method. The procedure may become a useful treatment option in patients who fail typical treatments, such as PTC or ERC for biliary complications after LDLT.

## Conclusions

We encountered a patient treated for post-LDLT biliary dehiscence by MCA. To our knowledge, this is the first case report of dehiscence of two bilioenteric anastomoses being treated by MCA after LDLT. MCA for stricture, obstruction, or dehiscence of the anastomosis after LDLT was found to be an effective and safe procedure in patients who could not be treated by conventional intervention.

## Abbreviations

BAS: Biliary anastomotic stricture
BL: Biliary leakage
ERC: Endoscopic retrograde cholangiography
LDLT: Living-donor liver transplantation
MCA: Magnetic compression anastomosis
MELD: Model for end-stage liver disease
PTC: Percutaneous transhepatic cholangiography
PTCD: Percutaneous transhepatic cholangiography drainage
QOL: Quality of life
RAD: Right anterior duct
RPD: Right posterior duct
